# An In Situ Study of Short Crack Initiation and Propagation During Fatigue Testing of a Hot Isostatically Pressed Al-7%Si-0.5%Mg (A357-T6) Alloy Specimen

**DOI:** 10.3390/ma17235928

**Published:** 2024-12-04

**Authors:** Toni Bogdanoff, Murat Tiryakioğlu

**Affiliations:** 1 Department of Materials and Manufacturing, Jönköping University, Box 1026, 55111 Jönköping, Sweden; toni.bogdanoff@ju.se; 2 School of Engineering and Technology, Jacksonville University, Jacksonville, FL 32216, USA

**Keywords:** bifilms, oxides, Si particle cracking, small crack growth, liquid metal damage

## Abstract

A hot isostatically pressed specimen of the A357 alloy in T6 condition has been tested for fatigue performance in situ. During testing, multiple small cracks were observed during the first cycle, both in proximity to and far from the stress concentration. These cracks have competed to form a propagating crack, forming multiple crack paths initially. Once the propagating crack has been established, it has chosen paths from multiple cracks that have opened around the tip to grow further. All small cracks observed to open have been attributed to bifilms, i.e., liquid metal damage. It is imperative to develop processes that minimize liquid metal damage to enhance the fatigue performance of aluminum alloy castings.

## 1. Introduction

It is well known that about 90% of in-service failures in engineered components are due to fatigue [1]. It is also widely accepted that there are three stages in fatigue failure: crack initiation (Stage I), crack propagation (Stage II) and final rupture (Stage III). In engineered components, total fatigue life is usually dominated by the number of cycles (N) in Stage I, where cracks are small, typically less than 1 mm [2]. Once a crack is initiated, its growth follows first the small crack regime and then propagates following the well-known Paris–Erdoğan [3] equation in Stage II until the final rupture (Stage III). When fatigue failures occur, it is common practice to determine where the fatigue crack was initiated ex situ, i.e., after failure. It is commonly assumed that cracks initiate only at one location, which is the culprit for fatigue failure. Crack initiators in aluminum alloy castings have been reported to be primarily pores and inclusions, such as oxides [4,5,6,7,8]. When large pores are not present, especially near the surface of the specimen as would be the case after a hot isostatic pressing (HIP) treatment, oxides [9] and intermetallic phase and/or second phase particles, such as the Si particles, in Al–Si alloys [10,11,12], act as crack initiators. Hence, finding the one crack initiator that has led to fatigue failure has been the most common practice.

However, recent research on in situ fatigue testing of cast aluminum alloys has shown that multiple cracks form during cyclic loading. Most cracks that have been observed to open during in situ tests are initially away from the propagating crack but have later coalesced with other cracks and finally joined with the propagating crack [13]. Similar observations have been made by Lee et al. [14] in their study on crack growth rates in cast Al–Si alloys, in which they have observed crack growth metallographically. These authors have stated that cracks are initiated in eutectic regions by either cracking or decohesion of the eutectic Si particles. Subsequently, the coalescence of microcracks has dominated the propagation stage in Si particles that have fractured or decohered, which are considered weak and brittle [15,16]. One of the authors and his coworkers [17] have observed that secondary small cracks have opened unexpectedly away from areas of higher stress. The authors have also reported similar findings [18] for an Al-5Si-0.5Mg-1Cu alloy casting. Characterization of these small cracks have shown that they are all oxide films formed in the liquid state.

Due to the high affinity of aluminum for oxygen, aluminum reacts with oxygen within nanoseconds [19]. These oxide films remain harmless as long as the surface is not interrupted. However, the current casting technology not only disrupts this surface multiple times but also causes the creation of new surfaces during pouring and mold filling. Consequently, these films fold over during liquid processing and mold filling to form two layers of oxides that have perfect bonding on one side and zero bonding between them. Campbell [20] has suggested that these bifilms form the Griffith crack. In an in situ study by X-ray tomography by Dahdah et al. [21,22], many cracks have been observed near as well as distant from the pores at the first cycle; the subsequent crack propagation has been simply the coalescence of many cracks between pores. Chan et al. [23] has reported that fatigue crack growth in B319 aluminum alloy specimens involved multiple cracks growing and later coalescing. These cracks that open with tensile stresses are almost certainly bifilms, as determined recently by Bogdanoff et al. [18]. Hence, there is growing evidence that fatigue crack initiation and growth is dominated by the degree of prior damage given to the liquid metal. This damage from the casting process has been mistakenly interpreted as fatigue damage in the literature.

In prior studies, either the small cracks that appear around pores and/or propagating cracks have been characterized or cyclic testing has been interrupted to determine the nature of small cracks. To the authors’ knowledge, an in situ study on the initiation of small cracks, their propagation, coalescence and growth leading to final fracture has not been reported in specimens without pores. This study using in situ testing is crucial for understanding how materials behave under working conditions and is motivated to fill this gap.

## 2. Experimental Details

An ingot of Al-7.1wt%-0.55wt%Mg alloy (A357) was melted in a boron nitride-coated crucible. The melt was then poured into a permanent copper die, pre-heated at 250 °C and coated with graphite to avoid molten metal and mold interaction. The cylindrical rods of 150 mm in length and 9 mm in diameter were remelted and drawn from a directional solidification furnace raising at approximately 6 mm/s. This technique produces a low-defect material because the solidification front can push inclusions toward the top of the samples [24]. The analysis of microstructure was performed with an Olympus optical microscope. Specimens were hot isostatically pressed (HIP), following the industrial practice, at 490 °C for 2 h with a pressure of 75 MPa to eliminate pores. Subsequently, they were solution-treated at 530 °C for 20 h and then quenched in water at 60 °C. After 24 h of natural aging, specimens were artificially aged at 190 °C for 5.5 h to reach the T6 condition.

The microstructure and fracture surfaces of the samples were examined using a TESCAN LYRA3 (Brno, Czech Republic) scanning electron microscope (SEM) coupled with energy dispersive spectrometry (EDS). Qualitative EDS point analysis was performed to determine the compositions of intermetallic phases on the fracture surfaces. The SEM was operated at acceleration voltages of 15 kV for all map measurements. Additionally, mapping of representative microstructure areas was conducted to reveal the distribution of phases. The EDS point analysis on the fracture surface was performed with an acceleration voltage of 5 kV.

Tensile testing specimens with a gauge length of 36 mm and a diameter of 6 mm were machined from the directionally solidified samples. Testing was conducted at room temperature following the ASTM E8 [25] standard with a constant crosshead speed of 0.5 mm/min. A total of five specimens were tested. A clip-on extensometer was used to measure the strain throughout the tensile test.

**Figure 1:**
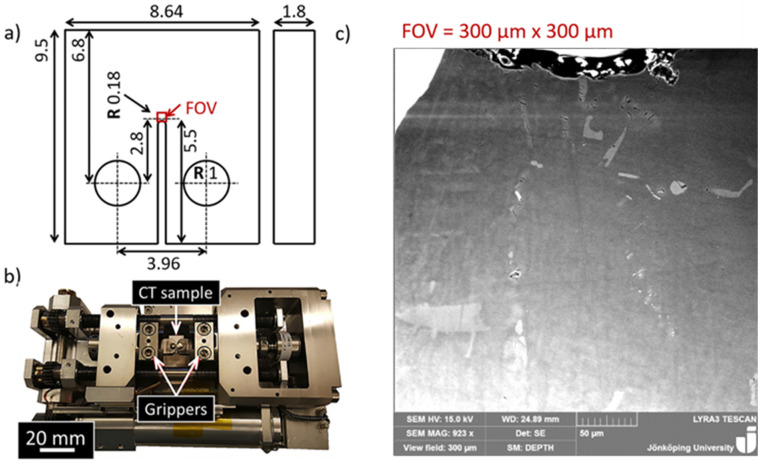
(**a**) Dimensions of the CT sample in mm [17]; (**b**) miniature stage for in situ cyclic tests [17]; (**c**) FOV of the A357 sample.

Miniature Compact Tension (CT) sample was cut using electric discharge machining with a 0.25 mm wire. The miniaturized CT sample dimensions, shown in Figure 1a, were designed based on the ASTM E647-00 [26] standard guidelines. CT samples were polished to produce a mirror finish for SEM observations. The field of view (FOV) covered the notch tip to investigate local strain development, with a size that was initially 300 μm × 300 μm, which, after a certain point in the experiments, was increased to 400 μm × 400 μm. In situ cyclic tests were performed on a tensile compression module (Kammrath & Weiss stage) (Figure 1b) inside the same SEM at room temperature. Before cyclic loading, the monotonic tension load to failure of the CT samples was performed to find the cyclic loading as presented in the earlier work. The load was set to 290 N with a constant load ratio (R) of 0.2. The speed of loading was 8 μm/s (~0.1 Hz). The preload view is presented in Figure 1c. A total of four specimens were tested.

## 3. Results and Discussion

### 3.1. Microstructure and Tensile Results

**Figure 2:**
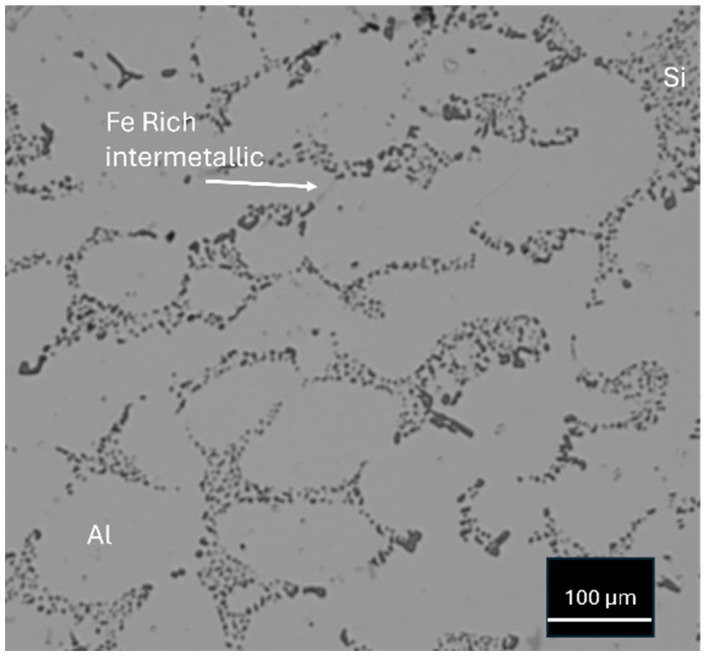
Microstructure of the A357 specimen. The arrow indicates a Fe-bearing intermetallic particle.

The final microstructure after HIP and heat treatment is presented in Figure 2 and is composed of Al α-dendrites and Al–Si eutectic particles. Moreover, Fe-bearing intermetallic phases have been observed. The secondary dendrite arm spacing was 60 ± 8 µm.

Tensile test results have shown that the material has a yield strength of 315 ± 4 MPa, a tensile strength of 347 ± 8 MPa and an elongation of 1.6 ± 0.5%. Based on the results from aerospace and premium quality castings [27], an elongation of 15.8% can be expected from this material as its ductility potential at a yield strength of 315 MPa. Hence, a quality index of 0.10 is calculated from the ratio of actual elongation to the ductility potential [28]. Because the quality index is below 0.25, it is concluded that the specimen has been highly damaged in its liquid state [29]. This result is noteworthy because no pores are left in the casting after the HIP process. Therefore, this specimen would pass all existing quality assurance tests, such as X-ray or even micro-CT scans, which rely solely on detecting pores to assess the quality of the casting. However, the damage remains hidden in the casting after HIP and becomes visible only when tensile stresses are applied, as demonstrated by in situ tensile tests recently [30,31]. Therefore, quality assurance practices that rely solely on detecting pores cannot assess the actual quality of the metal, as suggested recently [30].

### 3.2. Cyclic Testing

**Figure 3:**
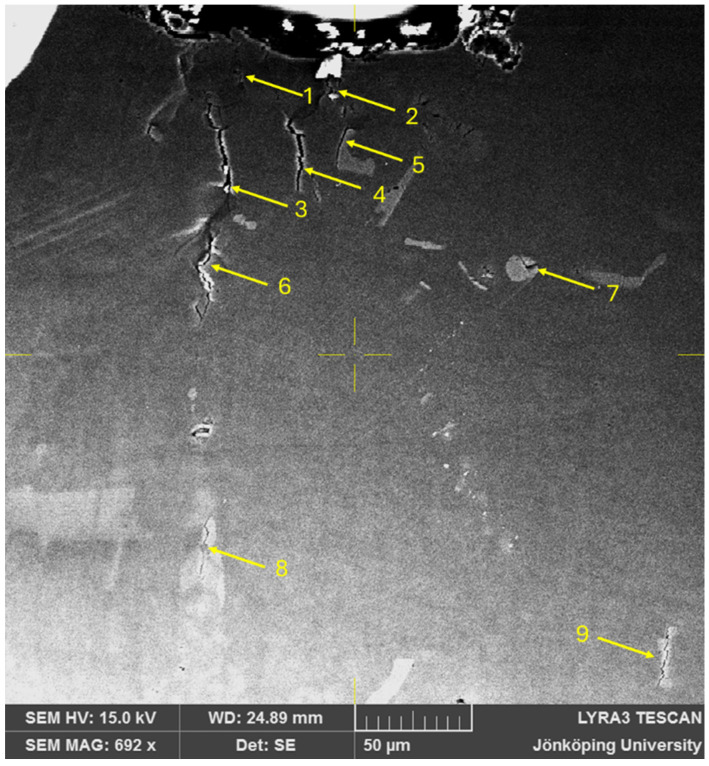
The specimen after the 1st cycle showed multiple cracks in the specimen and no crack connected to the stress concentrator.

On the very first cycle, multiple small cracks, with sizes on the order of 20 μm, have been initiated, as presented in Figure 3, which shows eight small cracks (features 1–6, 8 and 9) and a cracked Si particle (feature 7). Note that none of the cracks are connected to the surface where the stress concentrator is. It is also noteworthy that multiple cracks are in close proximity to the surface of the notch, namely cracks 1 through 5. Intense shear bands are visible between Cracks 3 and 6. Cracks 8 and 9 are far away from the stress concentration, where the effect of the stress concentration is not expected to be in effect.

It is significant that cracks appeared on the first cycle. It has been reported in several studies [18,32,33,34] on aluminum alloy castings that cracks start to grow from these defects almost at the first stress cycle of fatigue testing, as can be expected in metallic components with a high density of structural defects [35]. Since the HIP treatment has eliminated all pores, the only structural defects that can cause such cracks to open are bifilms. Multiple cracks appearing on the first cycle have not been observed before, to the authors’ knowledge. Five short cracks, 1 through 5 in Figure 3, can compete with each other to become the main propagating crack.

Another important aspect of the small cracks visible in Figure 3 is that they are associated with second phase particles. As noted above, Si eutectic particles have been found to crack and/or debond during cyclic testing. However, Si particles are also intrinsically strong. Mueller et al. [36,37] tested the fracture strength of Si particles in situ in an Al–Si alloy and found the fracture strength of these particles to be as high as 16 GPa when no defects were present in them. This is in perfect agreement with ab initio studies [38,39], in which an intrinsic strength of 16 GPa with a ductility of 30% was estimated for Si particles in an aluminum matrix. The strength of the Si particles was reduced only by extrinsic defects [40]. Additionally, the interfacial strength of the Si particles with the Al matrix is in the order of 7 GPa when no defects are present [41]. Similarly, Fe-bearing intermetallics have an intrinsic strength of approximately 19 GPa with an elongation of 14% [42,43,44]. Hence, the fracture of Si particles, their decohesion from the aluminum matrix and the fracture of Fe-bearing intermetallics are due to hidden entrainment defects, making them extrinsically weak. Bogdanoff et al. [18] have reported that Si and Fe-bearing particles that fractured in cyclic testing of a cast aluminum alloy had nucleated and grown on bifilms, which is consistent with previous results [45,46,47,48,49,50,51].

**Figure 4:**
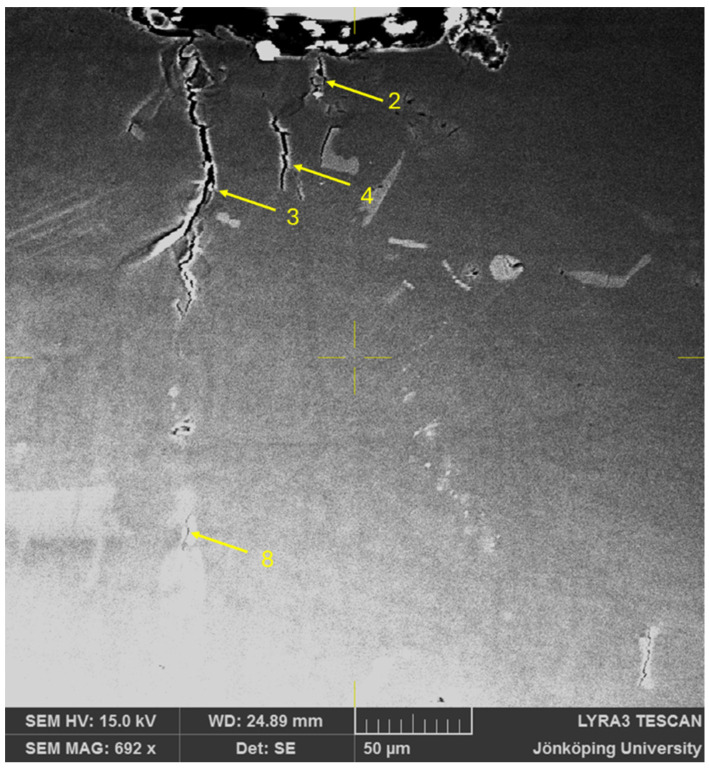
Cracks on the surface of the specimen after N = 16. Note that Cracks 3 and 6 in Figure 2 have now merged.

After sixteen cycles, Crack 3 has propagated toward the surface and reached it at the stress concentration, as presented in Figure 4. Hence, Crack 3 has won the competition between multiple cracks close to the stress concentration in Figure 3. Moreover, Cracks 3 and 6 have merged through propagation of both towards each other. Intense shear bands are still visible at their confluence. Crack 2 has also propagated toward the surface but has not reached it. Crack 4 has not propagated between the first and the 16th cycles. However, it is clear that a second path would be available for the main crack to develop through Crack 2 and then through Crack 4 if Crack 3 was less favorable. The presence of multiple cracks and propagation paths is analogous to an electrical circuit with multiple resistors in parallel. At the same maximum voltage (maximum tensile stress in fatigue testing), multiple resistors in parallel reduce the overall resistance and therefore a higher current passes through them. In fatigue, multiple crack paths reduce the resistance to crack propagation and increase the crack propagation rate.

**Figure 5:**
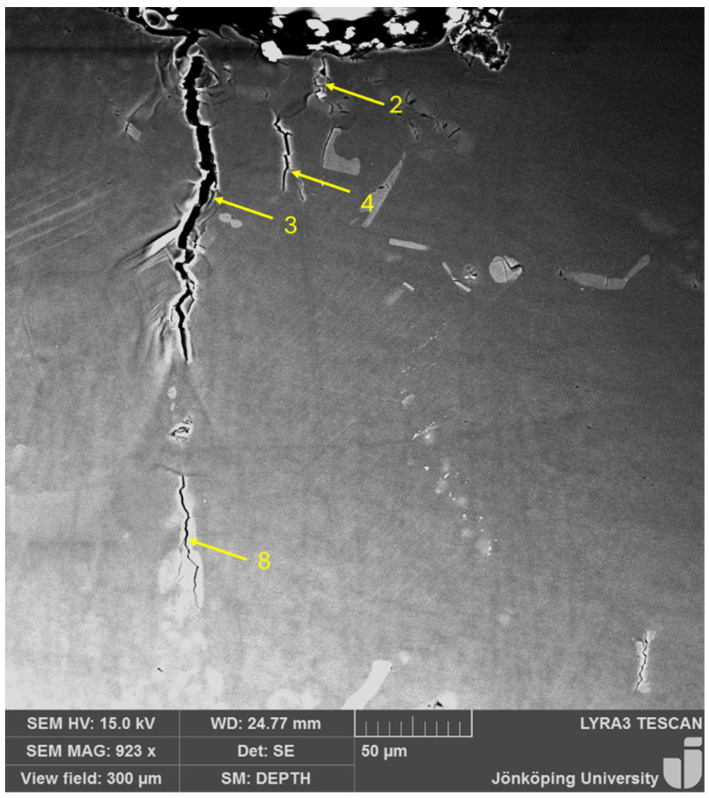
Cracks on the surface of the specimen after N = 100.

After 100 cycles, as presented in Figure 5 show that crack 3 has propagated downwards. A smaller side crack is visible slightly above the tip of the crack. Cracks 2 and 4 have not propagated at all. Crack 8, however, has started propagating toward Crack 3 and also further downward. Hence, multiple cracks have grown toward each other to merge to become a larger propagating crack. This has not been reported in the literature, to the authors’ knowledge.

**Figure 6:**
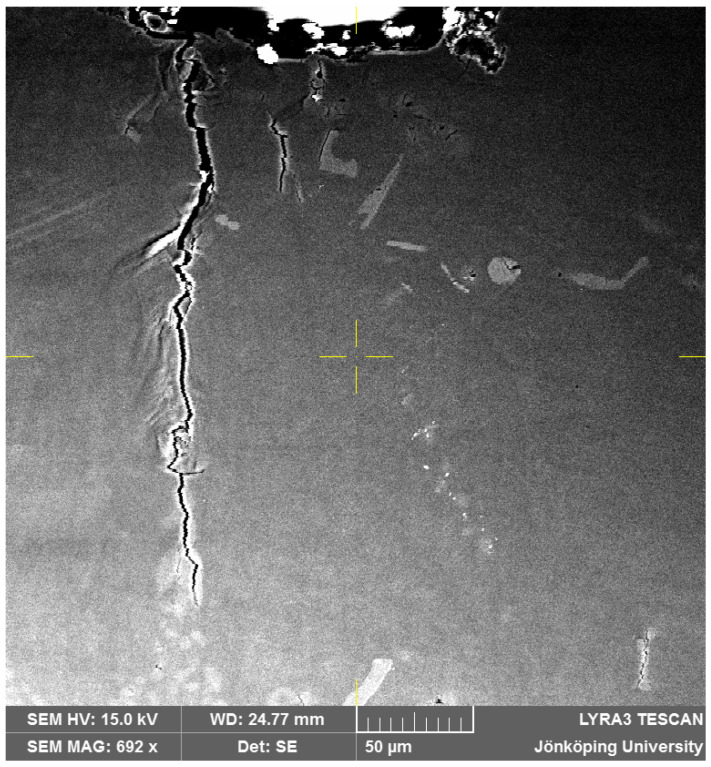
Cracks on the surface of the specimen after N = 143. Cracks 3, 6 and 8 in Figure 2 have coalesced.

The merging of Cracks 3 and 8 has been completed at N = 143, as shown in Figure 6. This crack has a length of approximately 200 μm and is still considered a short crack, which propagates downward along the eutectic region as indicated by many light gray eutectic particles. The opening of so many cracks in the first cycle and then the growth of multiple cracks to form a single propagating crack presents a scenario different from the common practice of determining one crack initiator that would be the culprit of the fatigue failure. The crack in Figure 5 results from the coalescence of three smaller cracks. It is also reasonable to assume that any forensic study after fracture would attribute the crack initiation site to the notch and not to these three crack initiation sites.

**Figure 7:**
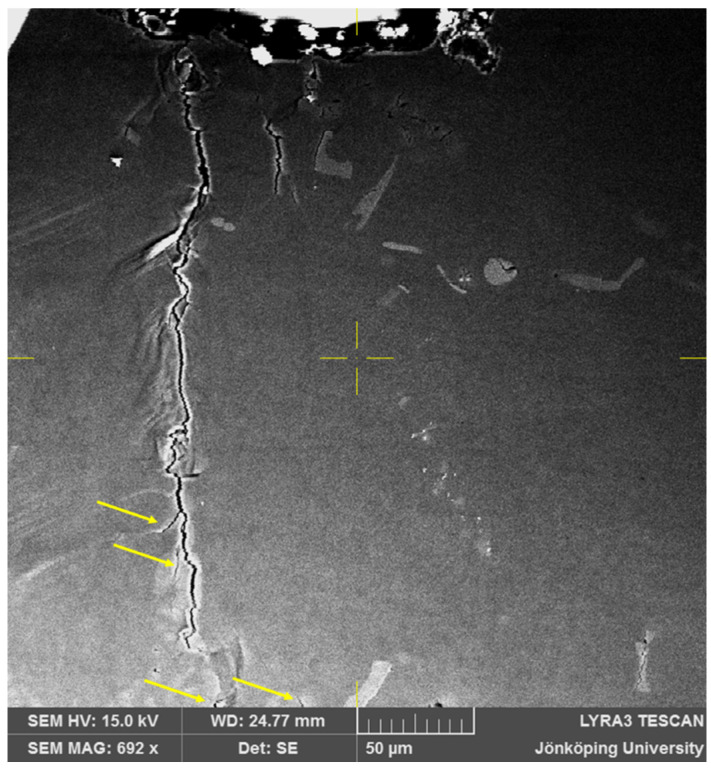
Cracks on the surface of the specimen after N = 215. Two side branches in the main crack and two new cracks ahead of the crack tip have become visible.

After 215 cycles, as seen in Figure 7, two side branches have become clearly visible, as indicated by arrows. Moreover, additional cracks, also indicated by arrows, have opened in front of the crack tip at the bottom of Figure 7. At this point, the FOV has been increased to 400 μm × 400 μm to continue to observe the crack growth behavior.

**Figure 8:**
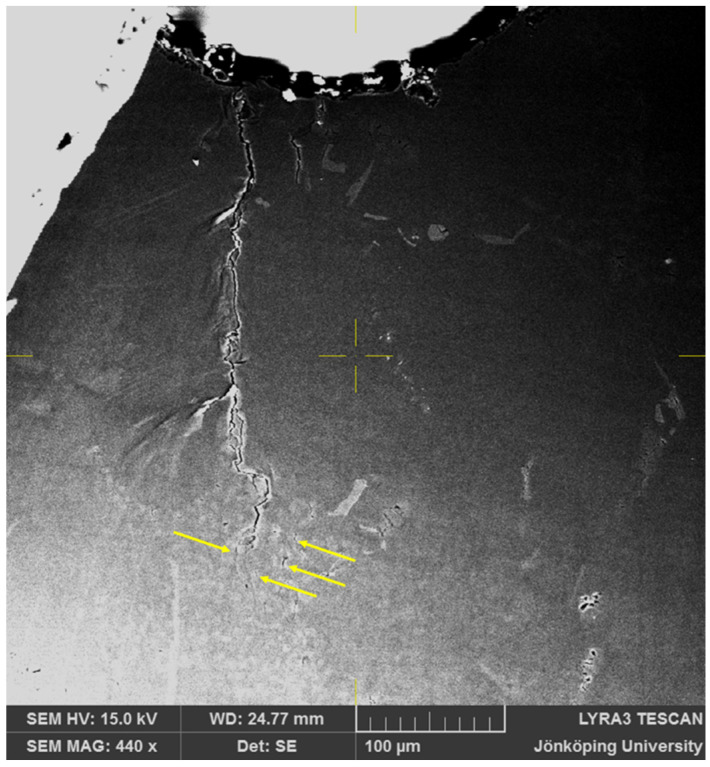
Cracks on the surface of the specimen after N = 254. Multiple cracks around the crack tip are readily visible.

**Figure 9:**
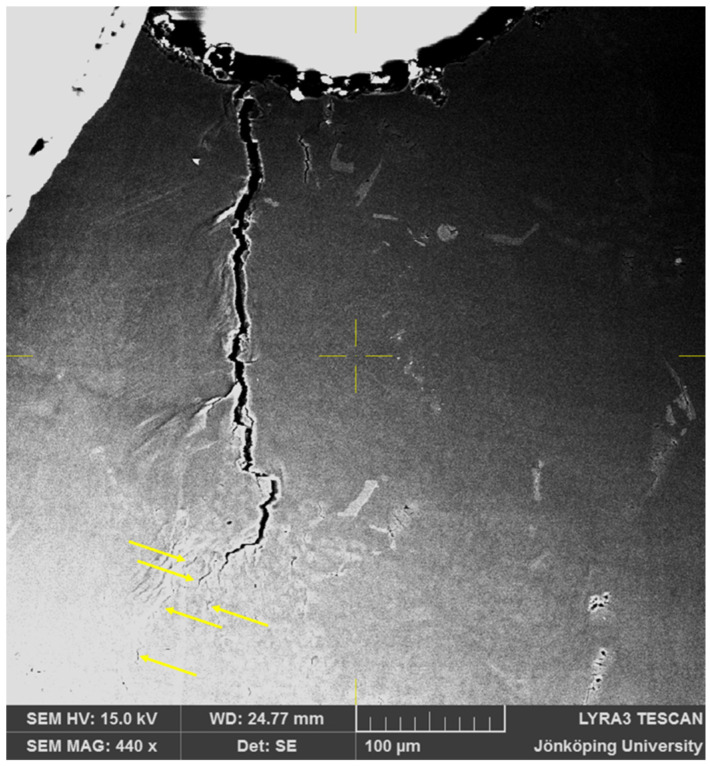
The crack and its tip at N = 300. Multiple cracks are opening around the crack tip.

**Figure 10:**
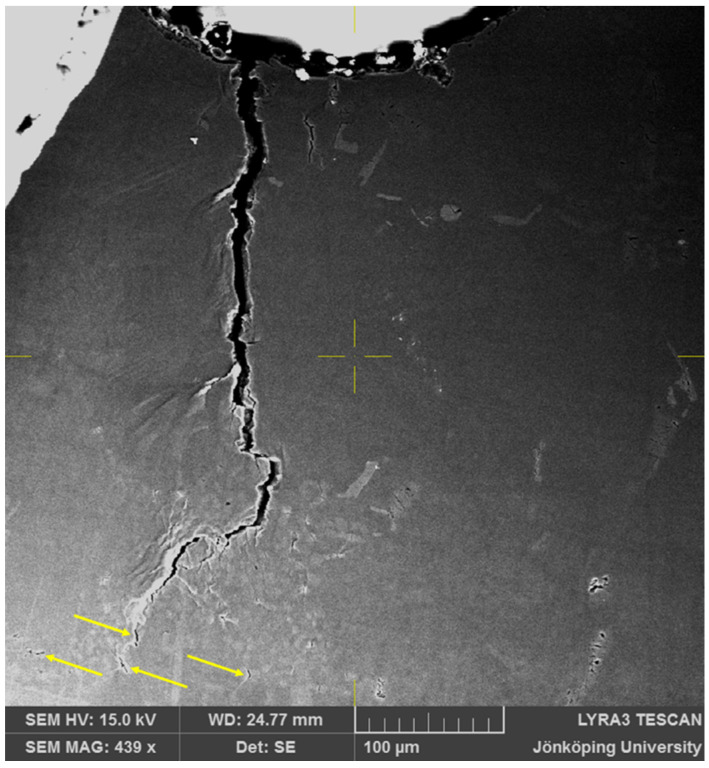
The crack and its tip at N = 310. Multiple cracks are opening up in front as well as on the right and left of the crack tip.

Figure 8 shows the crack path after 254 cycles. Since N = 215, the crack has taken two almost ninety-degree turns to go through one of the cracks that appeared at the bottom of Figure 7. Moreover, multiple new small cracks have appeared around the crack tip (indicated by arrows), giving it multiple options for further propagation. These new small cracks also seem to be associated with Si particles. The crack continues to grow, going through the cracks on the left side of the tip in Figure 8 as it reaches N = 300, as shown in Figure 9. As indicated by arrows, many more small cracks have now opened around the crack tip and one away from it. The crack propagates quickly through these existing cracks at N = 310, Figure 10. The crack has continued to follow the eutectic region, which seems to have provided less resistance to its growth. This is surprising because Shiozawa et al. [52] found that Si particles, as hard obstacles, deflected small cracks and reduced their growth rate in cast Al–Si alloys. This is in agreement with the computational results by Padkin et al. [53], who showed that particles with higher moduli of elasticity than the matrix should deflect the cracks, as would be the case in Si particles in an aluminum matrix. Similar results were reported by Gall et al. [12], who found that Si particles remained intact and served as barriers to crack growth in low-cycle testing of a cast Al–Si alloy. Lados et al. [54], however, determined that Si particle morphology played an important role, with large Si particles and/or those with a high aspect ratio provided less resistance to small crack growth, which was also reported by Spangerberg et al. [55] for A356 aluminum alloy castings. Dispinar and Campbell [56] found that Si particles that nucleated on bifilms were much coarser than those that nucleated homogeneously. Furthermore, the authors showed in an earlier work [18] using an SEM with a STEM analysis that the Si and Al_5_FeSi particles nucleated on bifilms. Hence, coarse Si particles are likely to have extrinsic defects through them, which would significantly lower the resistance to crack growth by cracking at lower stresses. This seems to be the case in the present study. If damage to liquid metal is minimized, Si particles can behave intrinsically and provide high resistance to fatigue crack growth, as reported by Shiozawa et al. [52].

**Figure 11:**
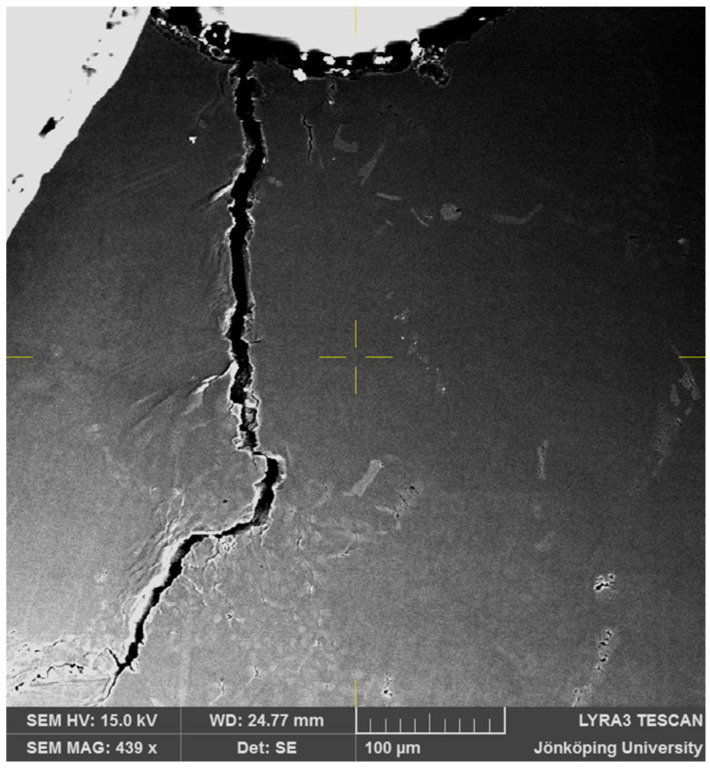
The crack and its tip just before the final fracture at N = 362. The crack tip has split into three branches.

The crack right before the final fracture at N = 362 is shown in Figure 11. Interestingly, the crack tip has split into three branches and has continued to go through the cracks that opened in front of the tip. To the authors’ knowledge, this split into three branches has not been reported before in the literature.

**Figure 12:**
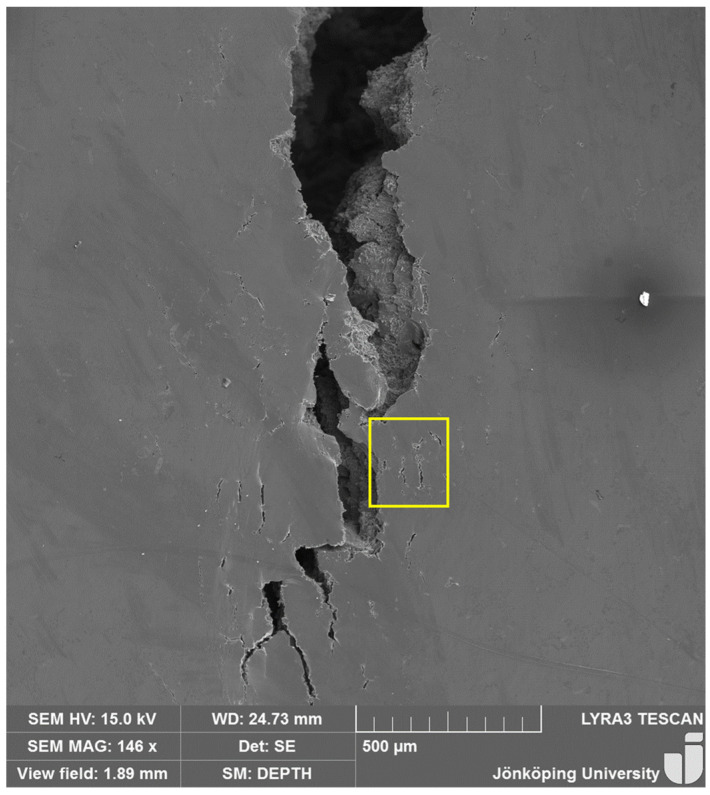
The crack after the final fracture.

**Figure 13:**
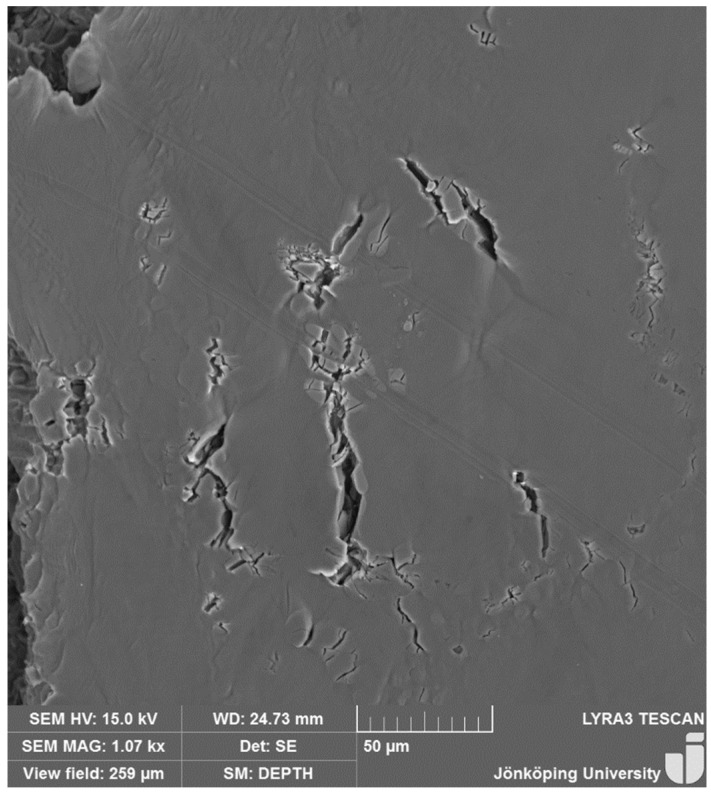
The details of the area within the box are in Figure 11. The small cracks seem to have taken place in the matrix as well.

The crack after the final fracture is presented in Figure 12. There are many smaller cracks that are open on the surface. The details of the area indicated by the box are presented in Figure 13. Note that aluminum matrix seems to have unzipped as well. Campbell [57] attributed such unzipping in a ductile matrix to bifilms, which, in this study, seem to have remained unbonded even after the HIP treatment.

### 3.3. Fractography

**Figure 14:**
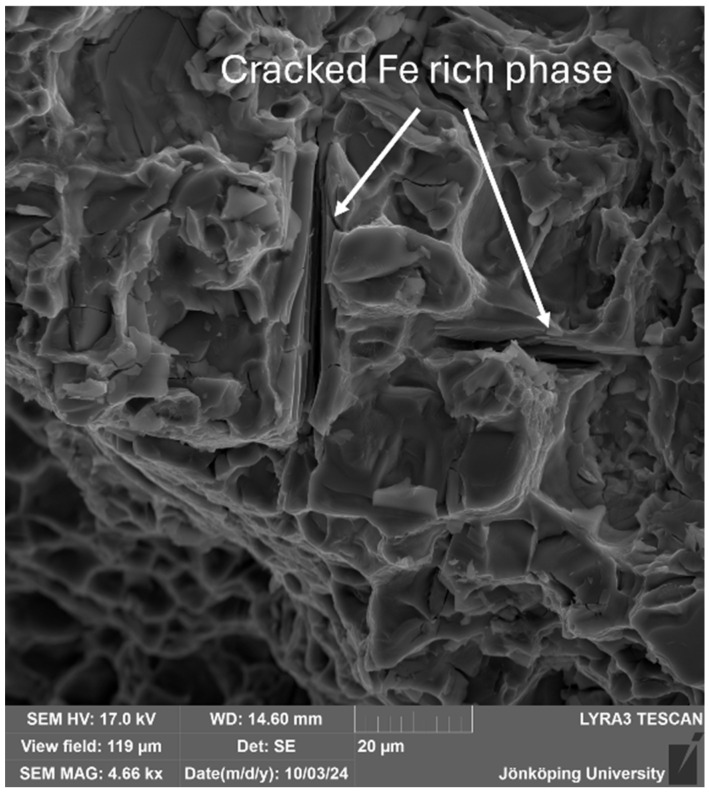
Cracked Fe-bearing particles were found on the fracture surface.

**Figure 15:**
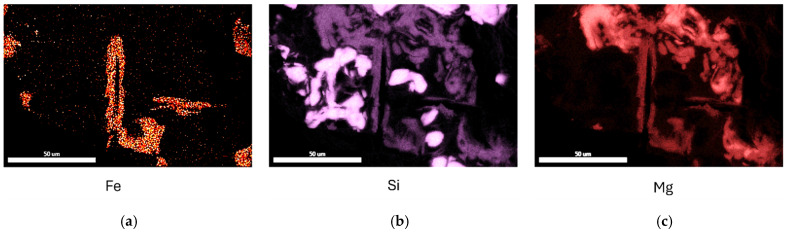
EDS maps for (**a**) Fe, (**b**) Si and (**c**) Mg in the same area as in Figure 14.

The fracture surface of the specimen has been investigated with an SEM. In Figure 14, cracked particles are visible, as indicated by arrows. X-ray maps for Fe, Si and Mg in the same area are presented in Figure 15. The coexistence of Fe, Si and Mg in the particles indicates that they are the π-Al_8_Mg_3_FeSi_6_ phase. This is consistent with the results by Ceschini et al. [58], who used specimens from the same batch of castings in their work. The fracture of the π-particles is noteworthy. Fe-bearing intermetallics are intrinsically strong and ductile [42,43,44], but they have also been shown to nucleate heterogeneously on bifilms [46,47,48,51,59]. Hence, their fracture in the middle can only be attributed to bifilms, which weaken the π-particles extrinsically.

**Figure 16:**
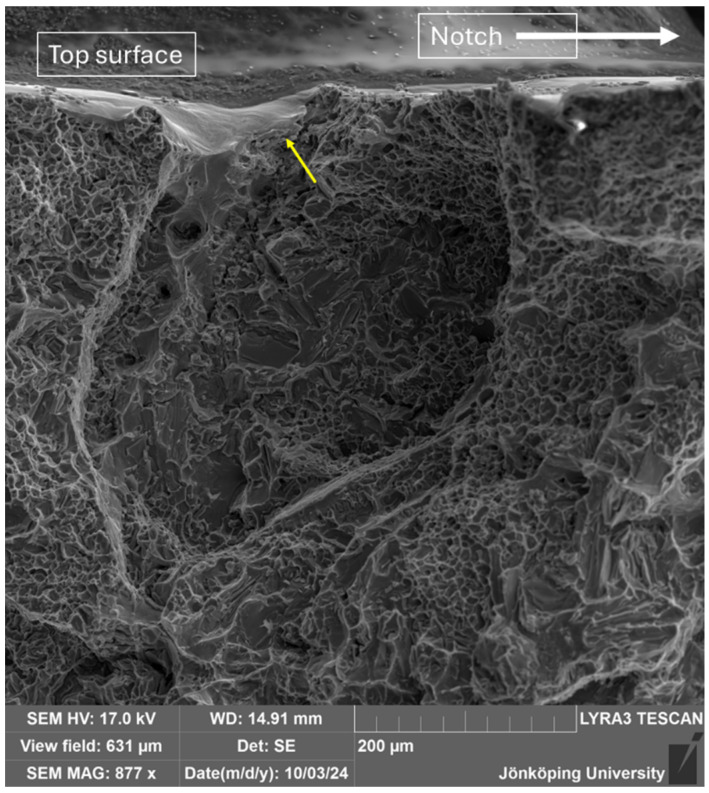
A feature on the fracture surface away from the notch.

**Figure 17:**
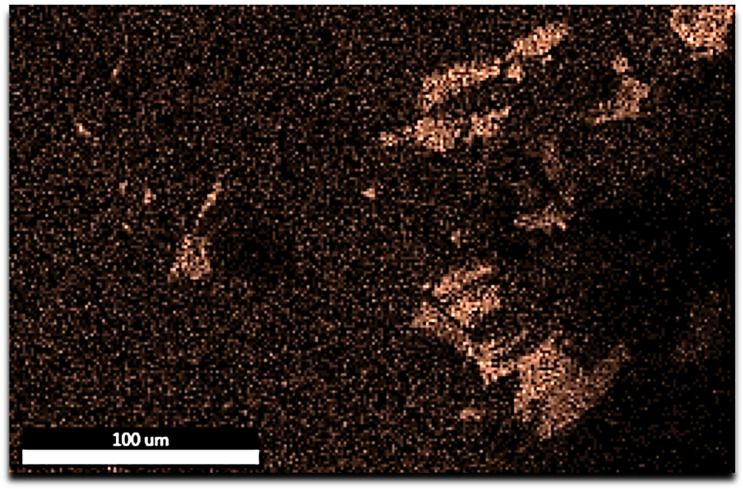
EDS map showing where Fe atoms are densely located.

Figure 16 shows an area that opened away from the crack tip during crack propagation. An oxide film resides at the top of this area, as indicated by the arrow. EDS maps near this oxide film have shown the presence of many π-particles in the region, as seen in Figure 17.

**Figure 18:**
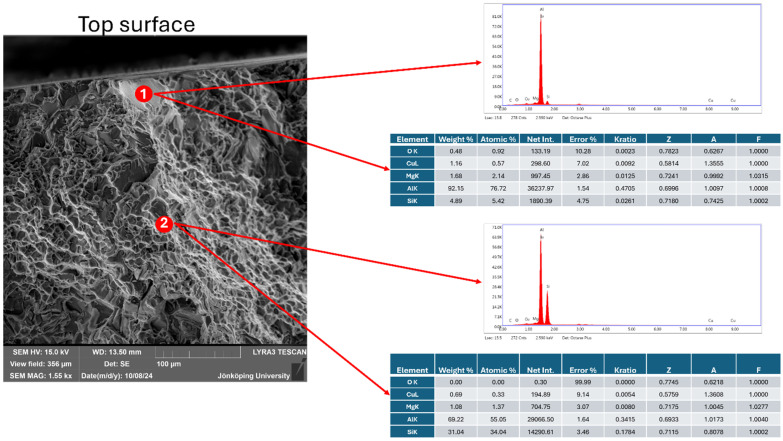
EDS point analysis of an area that opened during crack propagation.

Another area that opened during crack propagation is presented in Figure 18. The feature near the surface is usually referred to as a facet [60]. EDS point analysis of this area (indicated as 1) in Figure 18 has shown the presence of oxygen of approximately 1.0 at.%. However, in area 2, presented in Figure 18, no oxygen has been found. Hence, the facet near the surface of the specimen is clearly half of an oxide bifilm.

Based on the evidence provided in this study, liquid metal damage can remain hidden in the casting and survive all quality assurance procedures commonly used in the casting industry. However, the hidden liquid damage comes out during fatigue testing as multiple crack initiation sites and multiple paths for crack propagation, weakening the resistance of the microstructure to crack initiation growth and consequently, significantly reducing fatigue performance. Any forensic analysis of the fracture surface would attribute the poor fatigue performance to coarse Si particles and/or brittle Fe-bearing intermetallics. In reality, all cracks that have opened during this in situ study can be attributed to liquid metal damage. To increase the reliability of aluminum castings subjected to fluctuating loads, it is imperative to implement processes that minimize liquid metal damage in any stage of the casting production. Otherwise, aluminum castings will continue to pass nondestructive quality assurance tests and yet fail prematurely due to fatigue.

## 4. Conclusions

In this study, a hot isostatically pressed specimen, with no pores, was subjected to in situ fatigue testing. Multiple small cracks became visible in the first cycle near and away from the stress concentration. None of these cracks was connected to the stress concentration initially. Subsequently, there was a competition between these small cracks to become the main propagating crack. Some of these cracks coalesced and started propagating further, marking the end of the initial competition. This crack propagated through other pre-opened cracks. As the crack grew, multiple small cracks opened in front of it, providing many pathways to choose from, a phenomenon observed throughout this in situ test until the final fracture.

All cracks observed in this study were bifilms, created as a result of damage given to the liquid metal. Hence, liquid metal damage extrinsically reduced the fatigue resistance of the microstructure. Moreover, the presence of multiple small cracks and propagation paths was found to be analogous to resistors in parallel in electrical circuits, which reduced the overall resistance (to crack propagation in fatigue).

These results underline the misalignment between the production and quality assurance of aluminum castings. Castings without pores that would normally pass any quality assurance tests that rely on the detection of porosity can falsely conclude that a casting is sound when significant hidden damage exists in it. Therefore, it is imperative to implement processes that minimize liquid metal damage at any stage of casting production. Otherwise, aluminum castings will continue to pass nondestructive quality assurance tests and yet fail prematurely due to fatigue.

## Data Availability

The original contributions presented in the study are included in the article, further inquiries can be directed to the corresponding author.
